# Possible Internalization of an Enterovirus in Hydroponically Grown Lettuce

**DOI:** 10.3390/ijerph120708214

**Published:** 2015-07-17

**Authors:** Annalaura Carducci, Elisa Caponi, Adriana Ciurli, Marco Verani

**Affiliations:** 1 Laboratory of Hygiene and Environmental Virology, Department of Biology, University of Pisa, Via S. Zeno 35/39, 56127 Pisa, Italy; E-Mails: annalaura.carducci@unipi.it (A.C.); elicaponi@yahoo.it (E.C.); 2 Department of Agricultural, Food and Agro-Environmental Sciences, University of Pisa, Via del Borghetto 80, 56124 Pisa, Italy; E-Mail: ciurli@agr.unipi.it

**Keywords:** Coxsakievirus, viral food contamination, hydroponic culture

## Abstract

Several studies have shown that enteric viruses can be transferred onto the surface of vegetables and fruits through spray irrigation, but, recently, reports have suggested viral contamination of vegetables sub-irrigated with reused wastewater. Hydroponic cultures, used to grow ready to eat fresh lettuce, have also been used to study the possibility of viral absorption through roots. This study was conducted to assess a possible risk of viral contamination in lettuce from contaminated water. The leaves of lettuce plants grown in hydroponic cultures where the roots were exposed to water containing Coxsakievirus B2, were analysed for evidence of the virus. The plants and water were sampled at different times and virus was measured using quantitative RT-PCR and infectivity assay. In leaf samples, the lowest observed infective data were lower than the qRT-PCR detection limits, suggesting that free viral RNA or damaged viruses are eliminated rapidly while infectious particles remain stable for a longer time. The obtained data revealed that the leaves were contaminated at a water concentration of 4.11 ± 1 Log Most Probable Number/L (8.03 ± 1 Log GC/L) a concentration observed in contaminated untreated water of wastewater treatment plants. However, the absorption dynamics and whether the virus is inactive in the leaves still remains to be clarified. Nevertheless, this work has practical implications for risk management in using reclaimed water for agricultural use; when irrigated vegetables are destined for raw consumption, virological contamination in water sources should be evaluated.

## 1. Introduction

Epidemiological evidence indicates that enteric viruses are a growing cause of foodborne illnesses in developed countries [1]. In a 2015 report, the European Food Safety Authority found that 11.6% of cases of viral infections were transmitted by vegetables, fruit, berries, juices, and mixed food [2]. Foods that are eaten raw, such as vegetables, are frequently associated with outbreaks of gastroenteritis and hepatitis. Raw salad has been well documented in numerous outbreak reports and surveillance systems as a vehicle for foodborne viral infections [3,4,5,6]. In 2010, 11 outbreaks in Denmark were caused by various noroviruses that were spread on *Lollo Bionda* lettuce grown in France [5]. From 1992 to 2006 in England and Wales, noroviruses were associated with 17% of the foodborne illness outbreaks linked to salad vegetables and fruit [7]. In the United States, from 1991 to 2000 salad was implicated in 26% of the outbreaks of noroviral gastroenteritis [8], and, from 1990 to 2005, salads and lettuce contributed to 67% and 47%, respectively, of foodborne illnesses [9].

The prevalence of the norovirus genome in leafy greens coming from markets in Canada, Belgium, and France, in 2009 and 2010, were 28.8%, 33.3%, and 50%, respectively [10]. Although noroviruses are undoubtedly the pathogen most frequently responsible for foodborne infection caused by the consumption of vegetables, other viruses shed in the feces should not be ignored as they can be transmitted in a similar manner. In addition to Hepatitis A virus (HAV), which has also been implicated in vegetable related outbreaks [11], several studies suppose the same role for Enterovirus [12,13]. In particular, surveys have demonstrated a high viral occurrence in a variety of water environments suggesting that raw vegetables could be potentially contaminated with these viruses through irrigation water [14,15].

Vegetables can be contaminated with viruses at several points in the food chain [16], including during primary production [3,5] via water or manure, during manipulation by infected food handlers [4], or through cross contamination.

The quality of water used for irrigation is a crucial issue for the safety of horticultural crops [13]. Water can contaminate the surfaces of leaves, which has led to food production guidelines that recommend careful washing and sanitizing of food surfaces. However, many common treatments such as freezing, frozen storage, and washing with sanitizers, do not completely remove or inactivate enteric viruses [1,17,18,19]. This may be due to the plant tissues internalizing the viruses. Evidence of internalization has been previously documented for several pathogens, such as *Escherichia coli* O157:H7, salmonella, HAV, poliovirus, murine norovirus [20,21,22]. Several studies have demonstrated that enteric viruses can penetrate into plant crops through superficial cuts [23,24,25] or the stomata [26], or by absorption via root systems [27,28,29]. However, another study using water contaminated with poliovirus 1, adenovirus 40, Hepatitis A virus, and bacteriophages for sub-surface drip irrigation of cucumber and tomato plants, did not find virus in the aboveground parts of the plants [30].

Viral internalization depends on the specific virus [6,28], on transpiration, which is related to the relative humidity of the air, and on the virus concentration in the water [26]. 

Hydroponic cultures for the production of ready-to-eat salad have increased rapidly in recent years. This is a simple, convenient, and highly productive culture technique that allows farmers to obtain a clean product without soil residue. Virus internalization could pose a major risk of contamination in hydroponic cultures. The extended contact between the vegetable and water necessitates strict attention to water quality. However, existing regulations [13,31,32] consider only faecal bacterial indicators (*E. coli*) or pathogens (*Salmonella*, *Listeria*, *etc*.) for water monitoring purposes; they do not consider enteric viruses despite the fact that viral contamination is not related to bacterial parameters. The virological quality of water is important because most viruses have a low infectious dose and viruses are not effectively inactivated during post-harvest processing. 

Virological analysis of water samples requires that samples be concentrated and then analysed using polymerase chain reaction (PCR)-based methods [33]. There are several available concentration methods (ultrafiltration, ultracentrifugation, filtration, flocculation) [34] that have different recovery percentages. However, this technique only provides information about whether viral genomes are present, not about the viability of viruses. This analytical technique could be important for assessing the risk of biological contamination in vegetables and establishing concentration limits for the irrigation water. It is also important to address the viability of the virus comparing the biomolecular data with cell culture data.

In our study, we conducted a series of trials to evaluate the possible internalization and persistence of Enterovirus (Coxsakievirus B2), in lettuce plants grown in hydroponic cultures. As other enteric viruses chosen for the same aim by several authors [6,13], enteroviruses are pathogenic and small. This could facilitate their internalization through plant roots, and they have surface characteristics similar to noroviruses [35]. Moreover, with these viruses, the same sample can be analysed both with cultural and biomolecular methods without the use of surrogates, permitting the study of different inactivations of genome and infective particles at the same time and experimental condition.

## 2. Experimental Section

Lab scale experiments were conducted adding a viral suspension containing a known quantity of Coxsackievirus B2 to the water of hydroponic cultures of lettuce (*L. sativa*).

### 2.1. Virus and Cell Cultures

Coxsackievirus B2 was obtained from the Virology Unit of Pisa University Hospital. The virus was propagated in confluent monolayers of KB cells (subline of the ubiquitous KERATIN-forming tumor cell line HeLa) cultured in high-glucose Dulbecco’s modified Eagle’s medium (Invitrogen, USA) supplemented with 2% fetal bovine serum (Invitrogen, USA) at 37 °C in a 5% CO_2_ atmosphere. The virus was harvested two days post-inoculation by three freeze-thaw cycles and low speed centrifugation at 100 × *g* for 30 min. 

### 2.2. Hydroponic Cultures Preparation

**Figure 1:**
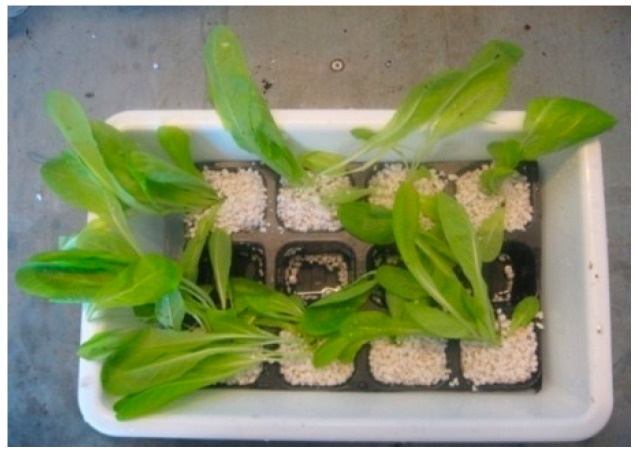
Hydroponic growth tank.

Hydroponic plants cultures were prepared in three phases: seeding, transplanting, and transferring. Monoclonal seeds were placed to cover the surface of autoclaved soil (Spezal Substrat, special composition) in plastic jars (nine-cm diameter). The seeds were sprayed with sterile water and incubated in growth chambers at 23 °C and 70% humidity with artificial lighting for 12 h per day. After the seeds had germinated and the germs had grown sufficiently, they were transplanted into pots filled with the same autoclaved soil. Three to four germs were placed at the centre of each pot and watered with 0.5 M Hoagland's nutrient solution [36]. The hydroponic tanks were lined with a non-woven plastic support material and filled with sterile vermiculite; 12 holes were drilled in the vermiculite. Next, the plants were taken from the pots and the roots were gently washed in sterile water to remove all soil residue. Than they were placed in vermiculite (one plant in each hole) and 1 L of sterile Hoagland’s solution was poured into each tank. The plants were grown in the laboratory under a fluorescent light cycle of 12 h light and 12 h dark for one week. The temperature and relative humidity were maintained at 23 °C and 70%, respectively (Figure 1). 

After this growing time, for the following experimental trials, the remaining eight tanks with 990 mL of nutrient sterile solution were prepared and, in each of them, five plants, prepared as above, were transferred inside.

### 2.3. Experimental Contamination

A suspension of Coxsackie B2 at a concentration of 11.62 Log GC/L (qRT-PCR) and an Most Probable Number of 11.30 Log MPN/L was introduced into the water solution of the hydroponic cultures. We also made two 10-fold dilutions of the virus suspension. In brief, 10 mL of the undiluted virus suspension or 10 mL of each dilution were mixed directly in the tanks containing 990 mL of nutrient sterile solution, avoiding contaminating the leaves. Two tanks were contaminated for each dilution and two were left as negative controls, by adding 10 mL of sterile water. 

At the same contamination time, one plant for each tank was sampled and treated as described below to evaluate if it was already contaminated at the starting time.

The expected final concentrations of the virus in the water of the tanks were 9.62 Log GC/L (9.30 Log MPN/L) for the lowest dilution and 7.62 Log GC/L (7.30 Log MPN/L) for the highest dilution. The plants were maintained for four days in the same culture conditions. All necessary precautions were taken to avoid contact between the water and the leaves, and not to contaminate the aboveground parts of the plants. 

Immediately after the virus suspension was added, 10 mL of contaminated water from each of the eight tanks were collected for analysis. On each of the following four days, 10 mL of solution and one plant from each tank were collected and run as independent biological replicates. For each day, we collected one sample for each tank corresponding to two replicates for viral dilution and negative control. 

Over the four days, a total of 40 water samples and 40 leaf samples were analysed. 

### 2.4. Water Sample Processing

The entire 10 mL contaminated water sampled from the tank was treated with chloroform (1 mL) to reduce bacterial contamination. The samples were agitated for 15 min, then centrifuged at 900 × *g* for 20 min at room temperature, aerated to eliminate the chloroform, and then the supernatant was recovered [37]. From each treated sample (10 mL), 140 µL was used for RNA extraction with the QIAamp Viral RNA Mini Kit (Qiagen, Hilden, Germany) into a final elution volume of 60 µL. Of the 10 mL sample, 860 µL were used for seeding the KB cell cultures for the MPN infectivity assay.

The estimated detection limits were considered as the minimum value of positive samples obtained by water analysis both with biomolecular than cell culture assays.

### 2.5. Plant Sample Processing

The method of processing plant samples was chosen through a preliminary series of tests using artificially contaminated samples that had known concentrations of viral suspensions spread on the leaf surfaces. These experiments also permitted one to evaluate the detection limits [38,39].

**Figure 2:**
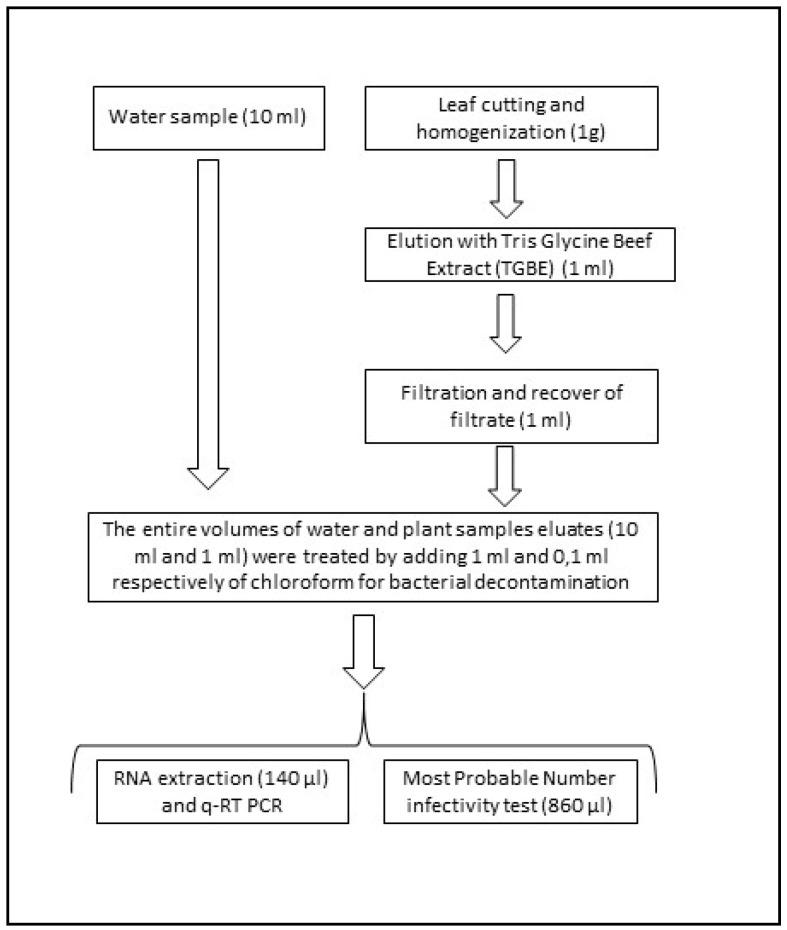
Sample processing for virus quantification. The sample volumes from each step are in parentheses.

To process the hydroponically grown test plants, the leaves were cut 1 cm above the root juncture, shredded, and homogenized with sterile scissors. For each sample, 1 g of shredded leaf was added to 9 mL of Tris-glycine-beef extract (TGBE; Tris-NaCl 100 mM, glycine 50 mM, and beef extract 3%) and shaken with two sterile steel balls at 500 rpm at 21 °C for 15 min. The sample was then centrifuged at 900 × *g* for 30 min at 4 °C. The supernatant was filtered with a 0.45-nm syringe filter and the filter was washed with 1 mL of TGBE. Each treated sample (1 mL) was decontaminated with chloroform (0.1 mL) and analysed using qRT-PCR and the MPN assay (Figure 2). 

### 2.6. Virus Quantification

#### 2.6.1. MPN Infectivity Tests

For the MPN tests, two 10-fold dilutions were prepared from each sample. The tests were performed in 96-well plates with KB cells monolayers and each sample dilution was tested in triplicate [40]. The plates were incubated in CO_2_ at 37 °C and read on the fifth day. The MPNs of infective viral particles were estimated on the basis of the number of wells showing a clear cytopathic effect. The final results were expressed as the number of Most Probable Number per Liter (MPN/L) for nutrient solution and the number of Most Probable Number per gram (MPN/g) for the leaves. 

##### qRT-PCR

For the qRT-PCR analysis, viral RNA was extracted using a commercial QIAamp Viral RNA kit (Qiagen, Germany). The concentration of Coxsackievirus B2 genomes was quantified by qRT-PCR using published protocols [41]; The primers in this assay (EVF: 5′-GGCCCCTGAATGCGGCTAAT-3′; EVR: 5′-CACCGGATGGCCAATCCAA-3′; EV Probe: 5′-FAM-CGGACACCCAAAGTAGTCGGTTCCG-TAMRA-3′) target the five-prime untranslated (5′-UTR) enterovirus region of 192 base pairs. The reactions, performed in triplicate, were conducted using Taqman one-step RT-PCR Master Mix (Applied Biosystems, USA). A standard curve was obtained using five dilutions, run in triplicate, from 10^6^ to 10^2^ genomic copies, of an amplicon (209 bp) from the 5’-UTR (Nanofab Scarl, Italy). 

For each series of samples, standard precautions were applied in all assays, including separate areas for the different steps of the protocol. In each qRT-PCR plate, a non-template control (NTC) and a positive control (known concentration of purified Coxsakievirus B2 RNA) were assayed in separate tubes. The presence of enzymatic inhibitors was evaluated by testing undiluted and 10-fold diluted extracted RNA for each sample. The extracted samples (10 µL) were analysed in 96-well optical plates. The plates were read with an ABI 7300 sequence detector system (Applied Biosystems, USA). The results were expressed as the number of genomic copies per liter (GC/L) for the nutrient solution and the number of genomic copies per gram (GC/g) for the leaves.

### 2.7. Data Analysis

We calculated the geometric means of the replicates for the qRT-PCR and infectivity MPN titres for each day and dilution. We used Graph Pad Prism statistical software (version 6) for data analysis. To calculate the geometric means and standard deviations taking in account the “negative” results, a viral load corresponding to half of the methods detection limit, was attributed to samples for which qRT-PCR and MPN were negative [42,43]. We also performed linear regression analysis between titres in the nutrient solution and leaves for both the qRT-PCR and MPN assay and between molecular and infectivity data, considering the singular matrix. 

## 3. Results and Discussion

### 3.1. Starting Plant Contamination

**Figure 3:**
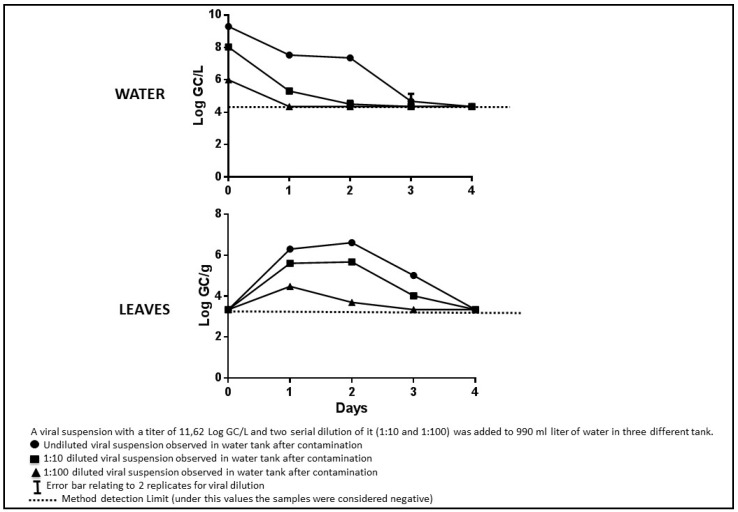
qRT-PCR logarithmic titers of water and leaves.

**Figure 4:**
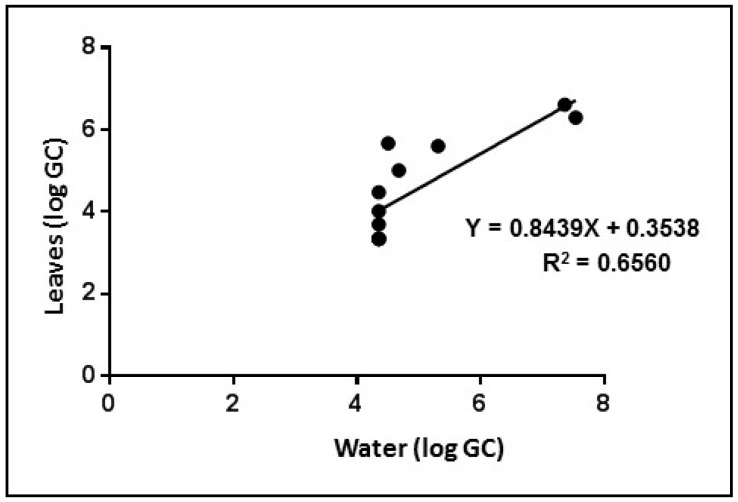
Linear regression analysis between water and leaves for qRT-PCR.

**Figure 5:**
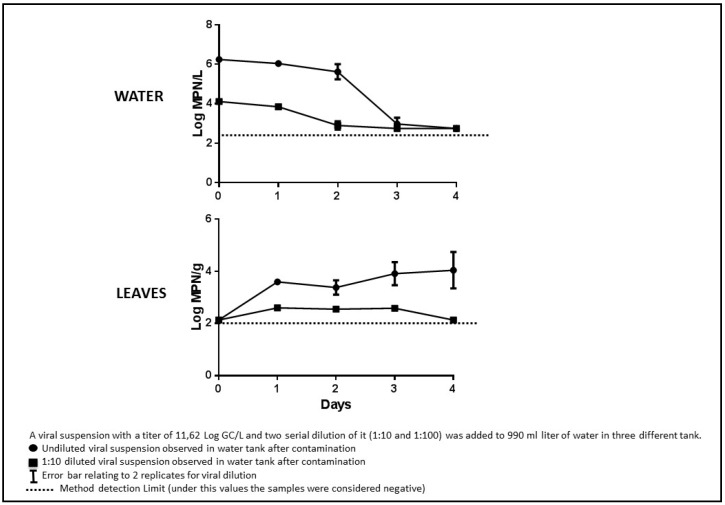
Cell Culture MPN logarithmic titers of water and leaves.

All the plants tested at the starting experiment time resulted negative for viral presence (Figure 3, Figure 4, Figure 5).

### 3.2. Starting Water Titre

According to the results of the qRT-PCR analyses, the viral titres of the water at the starting point of the experiment were similar to the expected values. We did not observe any contamination or enzymatic inhibition in any of the reactions. The viral titres based on the MPN assays at the starting point were lower than expected based on the amount of virus added. This could be due to adsorption of the virus onto the surfaces of the tanks, a phenomenon observed by other authors who evaluated different types of water and containers [23,35,44]. 

### 3.3. Methods’ Detection Limits

For water, the limits of detection were 4.65 Log GC/L for biomolecular tests and 3.05 Log MPN/L for cell culture assays. While for the plant treatment method, we obtained 3.65 Log GC/g and 2.01 Log MPN/g, respectively.

### 3.4. Experimental Tests

The mean viral titres measured using qRT-PCR in the water decreased from the initial concentrations during the course of the experiment, and no contamination or enzymatic inhibition were observed for any of the reactions (Figure 3). The control tanks resulted negative for each days tested. 

In the water contaminated with the undiluted virus suspension, which contained an observed average of 9.30 ± 1 Log GC/L at the beginning of the experiment, the virus decayed beyond the detection limit after four days. In water contaminated with a virus diluted to 8.03 ± 1.1 Log GC/L viral RNA, the observed titre was undetectable after three days, and in water contaminated with the virus at 6 ± 1 Log GC/L, the virus was undetectable on the first day. Viral contamination was detected in the leaves beginning on the first day at all three dilutions. The genomic copies inside the leaves remained stable for one day and then decreased on each of the following days. The decay observed in solution was also evident in leaves after the initial contamination. This could be attributable to the progressive elimination of virus integrity and RNA from exposure to vegetal fluids. Previous studies have reported that viral nucleic acids persist in the leaves for a short time [6,28]. We observed a significant positive correlation between the viral titres in the water and the leaves (R^2^ = 0.6560; *p* < 0.001) (Figure 4). 

The results from the cell culture titrations were not completely consistent with the qRT-PCR results (Figure 5). In the water samples, the virus was undetectable by the MPN assay at the 1:100 viral dilution in each analysed samples (not showed in figure). For the intermediate concentration (4.11 ± 1 Log MPN/L), the virus titre decreased from the first day and was undetectable on the third day. The virus concentration in the most contaminated water slightly decreased until the second day and then suddenly dropped below the limit of detection on the fourth day. In addition, in these experiments, the control tanks had negative results for each days tested.

**Figure 6:**
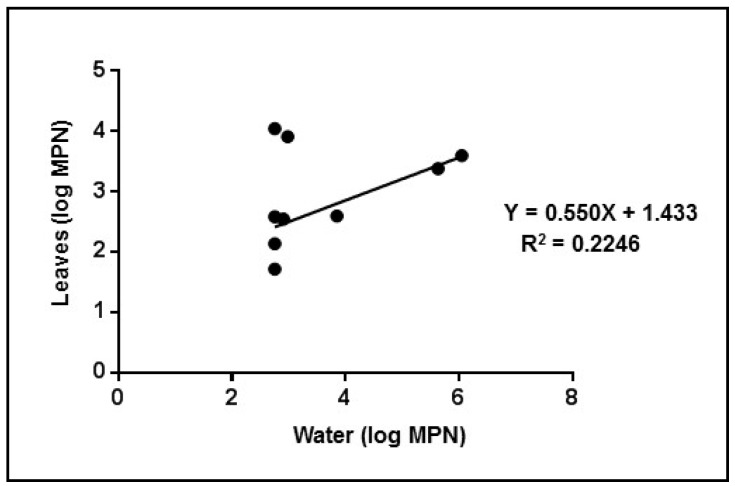
Linear regression analysis between water and leaves for cell culture MPN tests.

In the leaves, virus titres did not correspond exactly to the different levels of water contamination. No significant correlation was found between contamination of the water and leaves over time (R^2^ = 0.2246; *p* = 0.11); at the lowest virus dilution, we observed relatively stable viral titres in leaves, while the virus in the tank solution gradually disappeared. In fact, we observed positive results in the leaves even when the results were negative in the corresponding water sample. This could be due to adsorption of the virus onto the surfaces of the tanks but could also be attributed to plants protecting internalized viruses (Figure 6).

**Figure 7:**
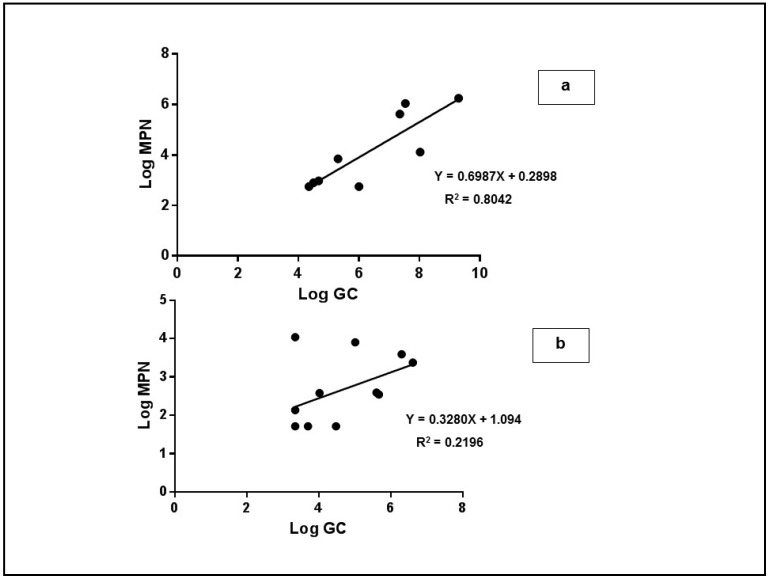
Linear regression analysis between qRT-PCR and cell culture MPN data for water (**a**) and leaves (**b**).

The linear regression analysis between the infectious virus and qRT-PCR titres for the water samples were positively correlated (R^2^ = 0.8024; *p* < 0.0001), but this was not observed in the leaves (R^2^ = 0.2196; *p* = 0.12) (Figure 7).

The lowest observed infectious virus titres in the MPN cell culture assay were lower than the qRT-PCR detection limits. Therefore, we hypothesize that, in the leaves, free viral RNA or damaged viruses are eliminated rapidly while infectious particles remain stable for a longer time. 

Our data indicated that the leaves were contaminated at a water concentration of 4.11 ± 1 Log MPN/L (8.03 ± 1 Log GC/L). This virus concentration has been observed in contaminated untreated water at wastewater treatment plants [31,45]. Furthermore, our results could suggest absorption of the enterovirus through the roots, since they are consistent with previous studies performed on other enteric viruses (human noroviruses and animal caliciviruses) in hydroponically grown plants [6].

## 4. Conclusions

Future studies are needed to clarify the possibility of roots’ absorption of enteroviruses, permitting the estimation of the minimum level of water contamination that does not contaminate the leaves and the persistence of viruses in the leaves. In addition, the localization of the internalized viruses should be studied using confocal microscopy or immunofluorescence techniques. 

Our results clearly indicate that evaluating viral contamination in reclaimed or surface water that is used for hydroponic cultures could be necessary for food safety. These results have practical implications for risk management regarding reclaiming water for agricultural use; namely when vegetables are destined for raw consumption, the virological contamination of the water used for irrigation should be evaluated.
